# Atypical Presentation of Severe Dengue in a Patient following a Major Abdominal Surgery

**DOI:** 10.1155/2020/2916107

**Published:** 2020-08-08

**Authors:** Umesh Jayarajah, Oshan Basnayake, Kavinda Nagodavithane, Jayan Jayasinghe, Dharmabandhu N. Samarasekera

**Affiliations:** ^1^Professorial Surgical Unit, National Hospital of Sri Lanka, Colombo, Sri Lanka; ^2^Department of Surgery, Faculty of Medicine, University of Colombo, Colombo, Sri Lanka

## Abstract

Severe dengue infections in a postoperative patient may lead to significant derangement in the body's homeostasis resulting in morbidity and sometimes even mortality. Reports on presentation and clinical manifestations of dengue in patients following major surgical procedures are scarce and restricted to few case reports. We describe a 26-year-old male with atypical presentation and late detection of dengue haemorrhagic fever following a major abdominal surgery. On postoperative day 6, he developed spontaneous bleeding from the drain site and moderate-to-massive bilateral pleural effusion with respiratory distress. His dengue IgM and IgG were positive. Therefore, a diagnosis of dengue haemorrhagic fever with bilateral lower zone pneumonia was made. A right-sided intercostal tube was inserted. Intensive care was given and was managed with intravenous antibiotics, targeted fluid therapy, and supportive care. He recovered from the infection and was discharged uneventfully. This case is unique because during the postoperative period, he went into critical phase with significant fluid leakage and developed bleeding manifestations without a clear febrile phase and deterioration in the haemodynamic parameters. High degree of suspicion and early detection are necessary to guide the fluid therapy and provide organ support in such patients.

## 1. Introduction

Dengue fever is a widespread arbovirus infection globally [1]. It is endemic in many developing countries including Sri Lanka and poses a considerable burden on healthcare systems [2, 3]. It has a spectrum of clinical manifestations ranging from subclinical infections to severe dengue with fluid leakage, haemorrhagic manifestations, and organ dysfunction [1]. Recent studies point to an increase in unusual presentations and severe disease [2, 4–7]. Severe dengue infection in a postoperative patient may lead to significant derangement in the body's homeostasis resulting in morbidity [8, 9]. Reports on presentation and clinical manifestations of dengue in patients following major surgical procedures are scarce and restricted to few case reports [9].

This case describes a unique occurrence of developing critical phase with significant fluid leakage without a clear febrile phase and unusual bleeding from the wound site following major abdominal surgery.

## 2. Case Presentation

A 26-year-old male with a history of subtotal colectomy and ileostomy for an inflammatory pelvic mass was referred to our unit for further assessment. He had no other medical comorbidities and had good exercise tolerance. Assessment with contrast enhanced computed tomography and magnetic resonance imaging showed resolution of the inflammatory mass, and a decision was made to restore the bowel continuity. Six months following the initial surgery, he underwent excision of the rectal stump (as it was found to be fibrotic) followed by an ileal pouch-anal anastomosis.

On the second postoperative day, he developed respiratory distress, tachycardia, and fever. Inflammatory markers were elevated with a white blood cell count (WBC) of 22 × 10^9^/l. Arterial blood gas showed a type 1 respiratory failure. A computed tomography (CT) pulmonary angiogram was performed suspecting a pulmonary embolism which was negative. However, there was bilateral basal consolidation. He improved with supportive care with resolution of fever within 24 hours.

**Figure 1:**
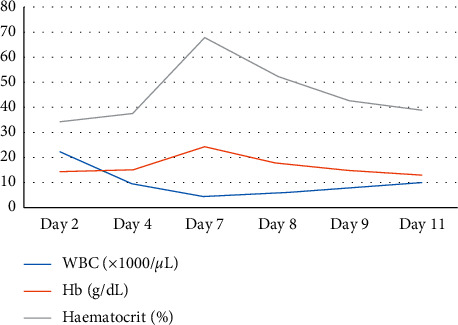
Image showing the trends of white blood cell, haemoglobin, and haematocrit with postoperative day.

**Figure 2:**
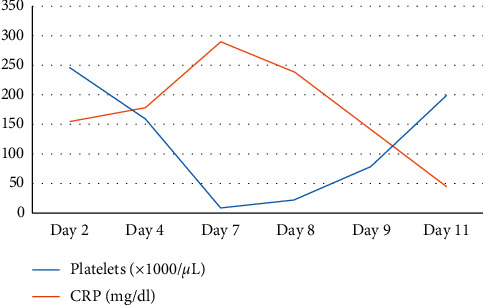
Image showing the trends of platelet and C-reactive protein with postoperative day.

**Figure 3:**
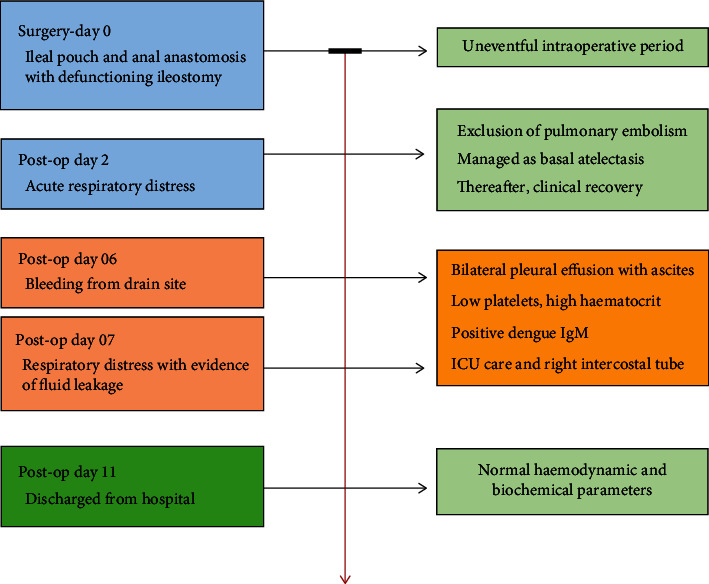
Timeline of key events and management.

Thereafter, his recovery was uneventful till day 5 with a functioning ileostomy and the abdominal drain was removed on day 4. On day 6, he had continuous bleeding from the drain site. On day 7, he again developed respiratory distress with a drop in saturation to 88% on air. There was drain-site oozing. He had no fever, and the haemodynamic parameters including urine output were normal. His full blood count revealed severe thrombocytopenia and haemoconcentration (WBC: 4.4, haemoglobin: 24.2 g/dl, haematocrit: 67.8, and platelet: 9), and C-reactive protein was 290 mg/dl (Figures 1 and 2). His liver enzymes were mildly elevated and renal functions were normal. His abdomen was nontender, and ileostomy was functioning normally. Ultrasound scan showed bilateral moderate-to-massive pleural effusion and moderate ascites. A right intercostal tube was inserted, and he was transferred to the ICU for intensive monitoring. The dengue IgM and IgG were positive. He was managed for dengue haemorrhagic fever and bilateral lower zone pneumonia with intravenous antibiotics, targeted fluid therapy, supportive care, and monitoring. Platelet transfusion was given as he had persistent drain-site oozing. He improved with supportive care. The drain site oozing seized after 48 hours, and the intercostal tube was removed on day 11. Thereafter, his recovery was uneventful and was discharged (Figure 3). Informed written consent was obtained from the patient prior to collection of data for publication.

## 3. Discussion

The evidence on presentation and clinical manifestations of dengue in postsurgical patients is restricted to case reports and case series and mostly includes transplant recipients [10]. In postoperative patients, bleeding from the surgical site and fever with abdominal symptoms are common presentations of dengue [10, 11]. However, these symptoms are nonspecific and, therefore, needs a high degree of suspicion for the diagnosis.

The reported patient developed respiratory distress and fever on day 2 after surgery which resolved within 24 hours. He did not have myalgia, arthralgia, or headache. The critical phase usually occurs towards the late febrile phase, often after the 3rd day of fever [1]. The reported patient had a brief period of fever on day 2 after surgery which resolved within 24 hours with improvement of clinical parameters.

He developed bleeding from the drain site on day 6 after surgery which may have been contributed by the use of nonsteroidal anti-inflammatory drugs given for pain relief. The critical phase was detected late once the patient developed respiratory distress due to bilateral pleural effusion. By then, there was severe thrombocytopenia and haemoconcentration. Full blood count was not observed after day 2 after surgery as the patient was clinically improving. The development of fluid leakage without a clear febrile phase was atypical leading to late detection.

The incubation period for dengue is generally 3–7 days (up to 12 days) [1]. As the patient was admitted 4 days prior to surgery, hospital acquired dengue is also a possibility. He did not have a blood transfusion before surgery excluding the possibility of transfusion-transmitted infection. This requires prompt investigation and implementation of preventive strategies in the hospital setting.

Following a major surgery, there is systemic inflammatory response due to the surgical stress and tissue trauma, challenging the homeostatic mechanisms in the body [12]. Additional insult due to a severe dengue infection may lead to further fluid shift and imbalance, microvascular instability, and compromise of homeostasis which may end up in morbidity or even mortality [1]. Therefore, preventive measures should be strictly implemented in the hospital setting in dengue-endemic areas to minimise postoperative morbidity.

## 4. Conclusion

We described a young male with atypical manifestation and late detection of dengue haemorrhagic fever following a major abdominal surgery. In dengue-endemic regions, unusual bleeding from a wound few days after surgery should raise a suspicion of dengue. Early detection is necessary to guide the fluid therapy and provide organ support during the critical period of illness. Preventive measures should be strictly adhered to minimise morbidity in postoperative patients.

## References

[1] McBride W. J. H., Bielefeldt-Ohmann H. (2000). Dengue viral infections; pathogenesis and epidemiology. *Microbes and Infection*.

[2] Jayarajah U., Faizer S., De Zoysa I. M., Seneviratne S. L. (2017). A large dengue epidemic affects Sri Lanka in 2017. *International Journal of Environmental Science and Technology*.

[3] Jayarajah U., de Silva P. K., Jayawardana P. (2018). Pattern of dengue virus infections in adult patients from Sri Lanka. *Transactions of The Royal Society of Tropical Medicine and Hygiene*.

[4] Jayarajah U., Seneviratne S. L., Gurugama P., Wanigasuriya K. (2017). Microalbuminuria and dengue viral infections. *The Southeast Asian Journal of Tropical Medicine and Public Health*.

[5] Gurugama P., Jayarajah U., Wanigasuriya K., Wijewickrama A., Perera J., Seneviratne S. L. (2018). Renal manifestations of dengue virus infections. *Journal of Clinical Virology*.

[6] Silva P., Jayawardena P., Jayawardena P. (2017). Improving clinical outcomes through setting up of a specialised dengue treatment unit. *International Journal of Advanced Research*.

[7] Jayarajah U., Dissanayake U., Abeysuriya V. (2020). Comparing the 2009 and 1997 World Health Organization dengue case classifications in a large cohort of South Asian patients. *The Journal of Infections in Developing Countries*.

[8] Jayasundara B., Perera L., de Silva A. (2017). Dengue fever may mislead the surgeons when it presents as an acute abdomen. *Asian Pacific Journal of Tropical Medicine*.

[9] Rawat S., Mehta Y., Juneja R., Trehan N. (2011). Dengue fever in a patient recovering from coronary artery bypass grafting. *Annals of Cardiac Anaesthesia*.

[10] Weerakkody R. M., Patrick J. A., Sheriff M. H. R. (2017). Dengue fever in renal transplant patients: a systematic review of literature. *BMC Nephrology*.

[11] Kumar S., Pushkarna A., Ganesamoni R., Nanjappa B. (2012). Dengue hemorrhagic fever as a rare cause of bleeding following percutaneous nephrolithotomy. *Urological Research*.

[12] Brøchner A. C., Toft P. (2009). Pathophysiology of the systemic inflammatory response after major accidental trauma. *Scandinavian Journal of Trauma, Resuscitation and Emergency Medicine*.

[13] Basnayake O., Jayarajah U., Jayasinghe J., Samarasekera D. N. (2019). Atypical presentation of severe dengue in a patient following a major abdominal surgery: a Case Report. *Journal of Gastroenterology and Hepatology*.

